# Knowledge and Perception of Cardiac Surgery Among Medical Students in the Western Region of Saudi Arabia

**DOI:** 10.7759/cureus.38605

**Published:** 2023-05-05

**Authors:** Osama A Abdulrahman, Rahaf G Baaqeel, Naif F Alotaibi, Raneem R Althebeti, Reem F Bahakeem, Omniyyah A Tukruni, Mohammad F Babgi, Abdulhalim S Serafi

**Affiliations:** 1 Faculty of Medicine, Umm Al-Qura University, Makkah, SAU; 2 Cardiac Surgery, King Abdullah Medical City, Makkah, SAU; 3 Department of Physiology, Faculty of Medicine, Umm Al-Qura University, Makkah, SAU

**Keywords:** perception, teaching, saudi arabia, knowledge, cardiac surgery, medical students

## Abstract

Introduction

Medical undergraduates' educational programs and clinical experiences are important factors in determining their preferred future career path. Unfortunately, the cardiac surgery specialty is experiencing a decline in medical graduates due to many influencing factors as a lack of involvement with the cardiac surgery specialty and a lack of training centers. A detailed evaluation of the student's knowledge and perceptions about cardiac surgery is required to assess the career options in a specialty like cardiac surgery. This study aims to evaluate medical students’ knowledge and perceptions of the cardiac surgical specialty.

Methodology

This is a cross-sectional study that was approved by the institutional research board of Umm Al-Qura University. Adapting a previously published questionnaire data to fit our scope and aims. Necessary adjustments were made adhering to the cardiac surgery experts' instructions. Data was collected through an electronic survey by Google Forms and distributed through social media apps.

Results

A total of 637 students participated in the study. The majority (75.2%) admitted to having little knowledge of the specialty of cardiac surgery, and 62.8% reported no interest in it. In addition, 88.9% had never done a cardiac surgery rotation before. One of the top concerns of becoming a cardiac surgeon (45.2%) was the amount of time spent studying and working.

Conclusion

The findings of our study highlight the value of using innovative and targeted learning methods for medical students to enhance their knowledge and pique their interest in cardiac surgery since it was evident that there was a misperception regarding the scope of cases dealt with by cardiac surgery as opposed to other surgical subspecialists.

## Introduction

The future career choices of medical students are influenced by the curriculum and exposure they receive in medical school. Medical students' interest in surgery may decrease due to a lack of exposure to surgical subspecialties throughout surgical rotations during medical school. However, most medical schools establish surgical interest groups to help students get more exposure to the field [1]. In 2005, Brundage et al. showed that 45% of first-year students desired surgical careers, but only 7% got matched with surgical residencies [2]. Cardiac surgery is a medical specialty that treats diseases of the heart. Factors that cause a decline in interest other than lack of exposure are the requirements to get into the residency program and the lack of knowledge about the future career of cardiac surgery [3]. Cardiothoracic surgery must be better exposed and accompanied by proper mentorship to fascinate more students [4]. An integrated six-year cardiothoracic surgery residency training program after medical school was developed to address the imminent shortage of surgeons, as well as streamline and improve core CT surgery training in contrast to traditional CT surgery residents, who have their entire general surgery residency program to explore CT surgery as a career choice [5]. Algethami et al. have shown that Saudi medical students have little interest in choosing cardiac surgery as their future career due to two primary factors, length of training and limited geographical locations of cardiac centers in Saudi Arabia [4]. The history of modern cardiac surgery, which began at the end of the 19th century, can be comprehended. Since then, cardiac surgery has evolved thanks to the efforts of many committed surgeons, who can now manage a wide range of cardiac pathologies. Today this process is still in progress. This activity covers recent advances in cardiac surgery and how minimally invasive surgery is altering the landscape of open-heart surgery [3]. A study showed that over the past decade, applications and interest in the surgical training pathway have plummeted, eventually affecting cardiac surgical specialties as well. And for various reasons, a significant number of medical students avoid cardiac surgery and decide to pursue other specialties instead [6]. A detailed evaluation of the student's knowledge and perceptions about cardiac surgery is required to assess the scope and career options in a specialty like cardiac surgery.

## Materials and methods

We designed a descriptive cross-sectional study that was approved by the institutional research board of Umm Al-Qura University (Approval No. HAPO-02-K-012-2022-09-1166) and conducted in the 2021-2022 academic year. This study targets undergraduate medical students in the western region from the third to the sixth year in nine different medical colleges (Al-Batterje, Faqeeh, Ibn Sina, Jeddah, King Abdulaziz, King Saud bin Abdulaziz, Taibah, Taif, and Umm Al-Qura). Consent was obtained from all study participants. All male and female medical students from the third to the sixth year of medical school in the Western Region of Saudi Arabia were included. We have excluded students who were ineligible or refused to participate, students from outside the Western region, and students in their first and second years because the first year is the foundation year and the second year is about basic science, and the third year also consider a basic year but they have a block of cardiovascular disease so we include them. The estimated number of medical students from third to the sixth years is 5000 full-time undergraduate medical students in the Western Region of Saudi Arabia. The minimum sample size required for this study to ensure a Confidence interval (CI) of 95% was calculated by OpenEpi version 3.0 to be 380 participants. Adapting previously published questionnaire data to fit our scope and aim [7]. Necessary adjustments were made adhering to the cardiac surgery experts' instructions. The questionnaire included 25 questions divided into the following sections: consent form, participants' demographics that included age, gender, region, place of residence, university, educational level, academic GPA, and nationality. Questions to assess their knowledge regarding the specialty of cardiac surgery, and their perception of the specialty, included areas of self-evaluation, interest in rotation, concern, cardiac surgery impression, the importance of the cardiac surgery department, source of information, and the financial and risk issues. and specific case scenarios to assess the knowledge regarding cardiac surgical procedures. Data was collected through an electronic survey by Google Forms and distributed through social media. A combined system of codes, numbers, and pseudonyms was set up to ensure the confidentiality of participants' information. Only researchers have access to the data. Data were analyzed using Statistical Package for the Social Sciences (SPSS) version 24 (IBM Corp., Armonk, NY, USA). Qualitative data were expressed as numbers and percentages, and the Chi-squared test (χ2) was applied to test the relationship between variables. A p-value of <0.05 was considered statistically significant.

## Results

A total of 637 students participated in the study, of which 51.0% were females and 49.0% were males. Of the participating students, 39.7% were citizens of Jeddah, 35.2% were students at Umm Al-Qura University, and 32.7% were in their sixth academic year (Table 1).

**Table 1:** Participants’ socio-demographic characteristics. (Total = 637)

Variables		No. (%)
Gender		
	Female	325 (51.0%)
	Male	312 (49.0%)
Place of accommodation		
	Jeddah	253 (39.7%)
	Madinah Al-Munawarah	82 (12.9%)
	Makkah Al-Mukarramah	168 (26.4%)
	Al-Qunfudhah	82 (12.9%)
	Taif	52 (8.2%)
University		
	Batterjee Medical College	67 (10.5%)
	Fakeeh College	17 (2.7%)
	Ibn Sina National college	33 (5.2%)
	University of Jeddah	83 (13.0%)
	King Abdulaziz University	49 (7.7%)
	King Saud bin Abdulaziz University	38 (6.0%)
	Taibah University	73 (11.5%)
	Taif University	53 (8.3%)
	Umm Al-Qura University	224 (35.2%)
Academic year		
	3rd year	123 (19.3%)
	4th year	170 (26.7%)
	5th year	136 (21.4%)
	6th year	208 (32.7%)
GPA out of 4		
	1.75 - 2.74	11 (3.9%)
	2.75 - 3.49	92 (32.3%)
	3.5 - 4	179 (62.8%)
	Less than 1	3 (1.1%)
GPA out of 5		
	2 - 2.74	4 (1.1%)
	2.75 - 3.74	39 (11.1%)
	3.75 - 4.49	158 (44.9%)
	4.5 - 5	151 (42.9%)

The majority of the participants have never taken a rotation in cardiac surgery (88.9%) and stated themselves as having little knowledge about it (75.2%); meanwhile only 8.8% considered themselves as having excellent knowledge about cardiac surgery. The most reported source of information was teaching sessions (35.2%), and the most chosen conditions to be treated by cardiac surgery were congenital heart defects, valvular insufficiency, and pacemaker implantation (71.4%, 62.2%, and 62%, respectively) (Table 2).

**Table 2:** Participants’ knowledge of cardiac surgery.

Variables	No. (%)
What is your knowledge about cardiac surgery?	
Excellent	56 (8.8%)
Little	479 (75.2%)
No knowledge at all	102 (16.0%)
Have you ever taken a cardiac surgery rotation?	
Yes	71 (11.1%)
No	566 (88.9%)
Which of the following conditions are treated by Cardiac Surgery (you can choose more than one)?	
Coronary artery disease	406 (36.3%)
Valvular insufficiency	396 (62.2%)
Aorta transaction	366 (57.5%)
Valvular stenosis	386 (60.6%)
Myocardial infarction	241 (37.8%)
Thoracic aortic aneurysm	339 (53.2%)
Lung cancer	102 (16.0%)
Left ventricular aneurysm	312 (49.0%)
Aortic dissection	377 (59.2%)
Diaphragm repair	169 (26.5%)
Congestive heart failure	195 (30.6%)
Pacemaker implantation	395 (62.0%)
Congenital heart defects	455 (71.4%)
What are the sources of information regarding cardiac surgery?	
Internet	148 (23.2%)
Television	23 (3.6%)
Friends	43 (6.8%)
Personal encounter	38 (6.0%)
Teaching session	224 (35.2%)
Clinical rotation	30 (4.7%)
Workplace	17 (2.7%)
Books	106 (16.6%)
Others	8 (1.3%)

The correlation between participants’ gender and their level of knowledge was with insignificant, but there was a significant association between their academic year and their level of knowledge (p-value = 0.018), as the sixth year students had the highest percentage of stating themselves as having an excellent knowledge (11.5%) and the lowest in stating that they have no knowledge at all (8.7%), in contrast to third year students whose 6.5% of them stated that they have excellent knowledge and 21.1% considered themselves to do not know (Table 3).

**Table 3:** Association between participants’ gender and academic year and their knowledge about cardiac surgery.

Variables		Excellent No. (%)	Little No. (%)	No knowledge at all No. (%)	P-value	
Gender	Female	23 (7.1%)	245 (75.4%)	57 (17.5%)	0.203	
	Male	33 (10.6%)	234 (75.0%)	45 (14.4%)		
Academic year	3rd year	8 (6.5%)	89 (72.4%)	26 (21.1%)	0.018	
	4th year	11 (6.5%)	126 (74.1%)	33 (19.4%)		
	5th year	13 (9.6%)	98 (72.1%)	25 (18.4%)		
	6th year	24 (11.5%)	166 (79.8%)	18 (8.7%)		

Cardiac surgery was the most reported specialty to deal with cases of pacemaker implantation, tetralogy of Fallot (TOF), coronary artery disease (CAD), and heart transplant (79.9%, 69.2%, 45.8%, 78.5%, respectively), and it was followed by interventional cardiology in the case of pacemaker implantation (28.1%), pediatric surgery in the case of TOF (48.2%), general cardiology in the case of CAD (44.0%). Cardiac surgery was the second most reported specialty following general cardiology (44.0%) in a case of severe chest pain consultation which counted for 34.4% and following vascular surgery (60.1%) in a case of abdominal aortic artery injury with 35.3%. In a case of a patient with punctured iron on his chest, thoracic surgery was chosen by 58.2% followed by cardiac surgery (41.6%) (Table 4).

**Table 4:** Participants’ responses to case scenarios related to cardiac surgery.

Variables		No. (%)
Case 1: A 30-year-old man need a pacemaker implantation which specialties do the implantation?	Vascular surgery	126 (19.8%)
	Pediatric surgery	18 (2.8%)
	Dermatologic surgery	14 (2.2%)
	Cardiac surgery (correct answer)	509 (79.9%)
	Orthopedic surgery	25 (3.9%)
	Plastic surgery	28 (4.4%)
	General surgery	68 (10.7%)
	Interventional cardiology (correct answer)	179 (28.1%)
	General cardiologist	119 (18.7%)
	Thoracic surgery	93 (14.6%)
Case 2: A baby born with Tetralogy of Fallot (CHD). Which specialties do the surgical repairs?	Vascular surgery	108 (17.0%)
	Pediatric surgery	307 (48.2%)
	Dermatologic surgery	18 (2.8%)
	Cardiac surgery (correct answer)	441 (69.2%)
	Orthopedic surgery	19 (3.0%)
	Plastic surgery	37 (5.8%)
	General surgery	42 (6.6%)
	Interventional cardiology	81 (12.7%)
	General cardiologist	68 (10.7%)
	Thoracic surgery	61 (9.6%)
Case 3: A 27-year-old male presents to the ER after a car accident with punctured iron on his chest. What specialties treat this type of injury?	Vascular surgery (correct answer)	133 (20.9%)
	Pediatric surgery	32 (5.0%)
	Dermatologic surgery	29 (4.6%)
	Cardiac surgery (correct answer)	265 (41.6%)
	Orthopedic surgery	79 (12.4%)
	Plastic surgery	52 (8.2%)
	General surgery	250 (39.2%)
	Interventional cardiology	76 (11.9%)
	General cardiologist	81 (12.7%)
	Thoracic surgery (correct answer)	371 (58.2%)
Case 4: A 45-year-old male presented to the ER with acute chest pain that investigation showed coronary artery disease which specialties would you refer him to manage his condition?	Vascular surgery	191 (30.0%)
	Pediatric surgery	23 (3.6%)
	Dermatologic surgery	21 (3.3%)
	Cardiac surgery (correct answer)	292 (45.8%)
	Orthopedic surgery	26 (4.1%)
	Plastic surgery	20 (3.1%)
	General surgery	53 (8.3%)
	Interventional cardiology (correct answer)	177 (27.8%)
	General cardiologist (correct answer)	280 (44.0%)
	Thoracic surgery	42 (6.6%)

Case 5: During your shift in the ER a patient was brought to the ER by his son that said his father had a severe chest pain and he was known with uncontrolled hypertension because he refused to take medication, which specialties you will consult?	Vascular surgery	122 (19.2%)
	Pediatric surgery	29 (4.6%)
	Dermatologic surgery	25 (3.9%)
	Cardiac surgery	219 (34.4%)
	Orthopedic surgery	28 (4.4%)
	Plastic surgery	18 (2.8%)
	General surgery	76 (11.9%)
	Interventional cardiology (correct answer)	139 (21.8%)
	General cardiologist (correct answer)	362 (56.8%)
	Thoracic surgery	42 (6.6%)
Case 6: A 15-year-old child with an end-stage-heart failure which need a heart transplantation which specialty could help in this management?	Vascular surgery	155 (24.3%)
	Pediatric surgery	105 (16.5%)
	Dermatologic surgery	23 (3.6%)
	Cardiac surgery (correct answer)	500 (78.5%)
	Orthopedic surgery	27 (4.2%)
	Plastic surgery	38 (6.0%)
	General surgery	84 (13.2%)
	Interventional cardiology	115 (18.1%)
	General cardiologist	165 (25.9%)
	Thoracic surgery	131 (20.6%)
Case 7: A 30-year-old male having a laparoscopic surgery, the surgeon by mistake injured the abdominal aortic artery, which the most important specialty should the surgeon call?	Vascular surgery (correct answer)	383 (60.1%)
	Pediatric surgery	22 (3.5%)
	Dermatologic surgery	23 (3.6%)
	Cardiac surgery	225 (35.3%)
	Orthopedic surgery	24 (3.8%)
	Plastic surgery	30 (4.7%)
	General surgery	142 (22.3%)
	Interventional cardiology	93 (14.6%)
	General cardiologist	57 (8.9%)
	Thoracic surgery	79 (12.4%)

69.2% considered cardiac surgery as an essential specialty, and 62.8% were not interested in it. Long years of study and hard work were the biggest concern about being a cardiac surgeon in 45.2% of the participated students. Students have expressed their thoughts about cardiac surgery, showing that 55.3% believed that open heart surgeries are always required in cardiac surgery, at the same time 65.3% reported that cardiac surgeries are not always necessary in treating heart problems, it also showed their thoughts about cardiac surgery not being the same as thoracic surgery (88.5%) nor the same as cardiothoracic surgery (61.2%). It was found that 91.1% thought that cardiac surgery operations are expensive and 89.5% thought of it as very risky (Table 5).

**Table 5:** participants' perception towards cardiac surgery.

Variables		No. (%)
What do you think about cardiac surgery as a specialty?	Essential	441 (69.2%)
	Like any other specialty	196 (30.8%)
Are you interested in cardiac surgery?	No	400 (62.8%)
	Yes	237 (37.2%)
What’s your biggest concern about being a cardiac surgeon?	Competitive specialty	130 (20.4%)
	Long years of study and hard work	288 (45.2%)
	Getting bored	66 (10.4%)
	No fears	122 (19.2%)
	Other	31 (4.9%)
Do you think cardiac surgery and thoracic surgery are the same?	No	564 (88.5%)
	Yes	73 (11.5%)
Do you think cardiac surgery and cardiothoracic surgery are the same?	No	390 (61.2%)
	Yes	247 (38.8%)
Do you think that cardiac surgery operations are expensive?	No	57 (8.9%)
	Yes	580 (91.1%)
Do you think cardiac surgeries are very risky?	No	67 (10.5%)
	Yes	570 (89.5%)
Do you think cardiac surgery always necessary to treat heart problems?	No	416 (65.3%)
	Yes	221 (34.7%)
Do you think open heart surgery always requires in cardiac surgery?	No	285 (44.7%)
	Yes	352 (55.3%)

There was a significant association between gender and academic year and their perception of cardiac surgery. Looking at students’ interest in cardiac surgery, out of the uninterested students 52.3% were males and 44.5% of them were sixth year students. 56.5% of the interested students were females and 40.5 of them were in their forth academic year (p-value = 0.032, p-value < 0.000, respectively) (Table 6, Table 7). Regarding cardiac surgery essentiality, sixth year students accounted for 39.8% of those who reported that is as essential as any other specialty (p-value = 0.016). Sixth year students were 42.6% of those who stated that they have no fears about being a cardiac surgeon and 51.6% of those who reported that they have other concerns about being a cardiac surgeon (Table 7).

**Table 6:** Association between participants' gender and their perception toward cardiac surgery.

Variables		Female No. (%)	Male No. (%)	P-value
What do you think about cardiac surgery as a specialty?	Essential	230 (52.2%)	211 (47.8%)	0.391
	Like any other specialty	95 (48.5%)	101 (51.5%)	
Are you interested in cardiac surgery?	No	191 (47.8%)	209 (52.3%)	0.032
	Yes	134 (56.5%)	103 (43.5%)	
What’s your biggest concern about being a cardiac surgeon?	Competitive specialty	67 (51.5%)	63 (48.5%)	0.750
	Long years of study and hard work	140 (48.6%)	148 (51.4%)	
	Getting bored	38 (57.6%)	28 (42.4%)	
	No fears	64 (52.5%)	58 (47.5%)	
	Other	16 (51.6%)	15 (48.4%)	
Do you think cardiac surgery and thoracic surgery are the same?	No	296 (52.5%)	268 (47.5%)	0.040
	Yes	29 (39.7%)	44 (60.3%)	
Do you think cardiac surgery and cardiothoracic surgery are the same?	No	221 (56.7%)	169 (43.3%)	<0.000
	Yes	104 (42.1%)	143 (57.9%)	
Do you think that cardiac surgery operations are expensive?	No	29 (50.9%)	28 (49.1%)	0.982
	Yes	296 (51.0%)	284 (49.0%)	
Do you think cardiac surgeries are very risky?	No	28 (41.8%)	39 (58.2%)	0.110
	Yes	297 (52.1%)	273 (47.9%)	
Do you think cardiac surgery always necessary to treat heart problems?	No	208 (50.0%)	208 (50.0%)	0.480
	Yes	117 (52.9%)	104 (47.1%)	
Do you think open heart surgery always requires in cardiac surgery?	No	149 (52.3%)	136 (47.7%)	0.567
	Yes	176 (50.0%)	176 (50.0%)	

**Table 7:** Association between participants' academic year and their perception toward cardiac surgery.

Variables		3rd year No. (%)	4th year No. (%)	5th year No. (%)	6th year No. (%)	P-value
What do you think about cardiac surgery as a specialty?	Essential	93 (21.1%)	128 (29.0%)	90 (20.4%)	130 (29.5%)	0.016
	Like any other specialty	30 (15.3%)	42 (21.4%)	46 (23.5%)	78 (39.8%)	
Are you interested in cardiac surgery?	No	57 (14.2%)	74 (18.5%)	91 (22.8%)	178 (44.5%)	<0.000
	Yes	66 (27.8%)	96 (40.5%)	45 (19.0%)	30 (12.7%)	
What’s your biggest concern about being a cardiac surgeon?	Competitive specialty	33 (25.4%)	40 (30.8%)	27 (20.8%)	30 (23.1%)	0.041
	Long years of study and hard work	54 (18.8%)	84 (29.2%)	61 (21.2%)	89 (30.9%)	
	Getting bored	12 (18.2%)	18 (27.3%)	15 (22.7%)	21 (31.8%)	
	No fears	18 (14.8%)	25 (20.5%)	27 (22.1%)	52 (42.6%)	
	Other	6 (19.4%)	3 (9.7%)	6 (19.4%)	16 (51.6%)	
Do you think cardiac surgery and thoracic surgery are the same?	No	111 (19.7%)	146 (25.9%)	116 (20.6%)	191 (33.9%)	0.160
	Yes	12 (16.4%)	24 (32.9%)	20 (27.4%)	17 (23.3%)	
Do you think cardiac surgery and cardiothoracic surgery are the same?	No	87 (22.3%)	88 (22.6%)	80 (20.5%)	135 (34.6%)	0.006
	Yes	36 (14.6%)	82 (33.2%)	56 (22.7%)	73 (29.6%)	
Do you think that cardiac surgery operations are expensive?	No	17 (29.8%)	10 (17.5%)	10 (17.5%)	20 (35.1%)	0.108
	Yes	106 (18.3%)	160 (27.6%)	126 (21.7%)	188 (32.4%)	
Do you think cardiac surgeries are very risky?	No	16 (23.9%)	17 (25.4%)	12 (17.9%)	22 (32.8%)	0.735
	Yes	107 (18.8%)	153 (26.8%)	124 (21.8%)	186 (32.6%)	
Do you think cardiac surgery always necessary to treat heart problems?	No	80 (19.2%)	109 (26.2%)	83 (20.0%)	144 (34.6%)	0.454
	Yes	43 (19.5%)	61 (27.6%)	53 (24.0%)	64 (29.0%)	
Do you think open heart surgery always requires in cardiac surgery?	No	59 (20.7%)	70 (24.6%)	61 (21.4%)	95 (33.3%)	0.690
	Yes	64 (18.2%)	100 (28.4%)	75 (21.3%)	113 (32.1%)	

## Discussion

Medical undergraduates' educational programs and clinical experiences are important factors in determining their preferred future career pathway. Thus, our goal was to evaluate Saudi Arabian medical undergraduates' knowledge in the field of cardiac surgery.

Six hundred thirty-seven medical students were included. The majority of the participants (75.2%) admitted to having little knowledge of cardiac surgery, and 62.8% reported no interest in it. In addition, 88.9% had never done a cardiac surgery rotation before, which was consistent with the findings of a previous study conducted in Saudi Arabia [4]. One study used session-based surveys of common cardiac surgery topics like extra-corporal membrane oxygenation (ECMO) and cardiopulmonary bypass (CBP) to evaluate the effectiveness of cardiac surgery mini-elective sessions in improving undergraduates' knowledge about the specialty. The study found a significant improvement when comparing the results of pre- and post-session surveys [8]. The results of a similar study showed an 81% rise in interest in the field of cardiac surgery [3]. Following participation in a simulation curriculum, there was a noticeable increase in interest in cardiac surgery. As part of the curriculum, students simulated cardiothoracic surgical procedures like large vessel anastomosis, open thoracotomy, and heart transplant [9,10]. It is advised to increase undergraduate curriculum exposure to cardiac surgery using various approaches, such as simulated sessions and survey-based sessions, in light of our alarming findings and the results of prior publications.

As per our results, 48.2% of students selected pediatric surgery as the appropriate specialty for treating a neonate with congenital heart disease, it was evident that there was a misperception regarding the extent of cases dealt with by cardiac surgery as opposed to other surgical subspecialists. Additionally, General Surgery was selected by 39.2% of respondents as the appropriate specialty for managing chest injuries necessitating cardiothoracic surgical intervention. Moreover, 34.7% of respondents believe that cardiac surgery is the only method for treating all forms of heart disease, and more than half (55.3%) think an open technique is always necessary for cardiac surgery. As a result, increasing medical students' exposure to the subject of cardiac surgery throughout the medical school curriculum may pique their interest in the field and clear up any misconceptions they may have about it.

To create an integrated generation of surgeons, summer elective training in Saudi Arabia needs to be better organized and concentrated on simultaneously motivating and educating students. One study sought to attract the attention and interest of medical students in Cardiothoracic Surgery by developing an eight-week summer training program that included an introduction, planned attachments for students to shadow during surgical interventions and in the clinic, as well as opportunities for students to participate in laboratory research and finally present their projects at the end of the training program. This initiative has yielded a significant increase in students' interest with 80% of eligible participants for residency programs having pursued a career in a surgical specialty [11].

One of the top concerns of becoming a cardiac surgeon (45.2%) was the amount of time spent studying and working. Our findings were in line with those of a Saudi Arabian study in which medical students reported that the long program years and lack of training facilities were the main deterrents to choosing cardiac surgery [4]. In comparison to another study, different results were observed, as job availability was the top concern for general surgery residents, and the main influencing factor was early exposure to cardiothoracic surgery specialty and the presence of good quality mentorship [12], implying that participants' educational level influences their choices.

There was no significant difference in the level of knowledge about cardiac surgery and gender, which matched a previous study that found no significant differences in interest in Cardiothoracic Surgery between genders. However, because they were less likely to get married or have children, women were more likely than men to experience social life disabilities, especially in a field like cardiac surgery [13-15]. In contrast to previous studies that assessed medical undergraduates' knowledge of plastic surgery, females were more knowledgeable than males due to their greater interest in cosmetics [7]. The findings of our study highlight the value of using innovative and targeted learning methods for medical students to enhance their knowledge and pique their interest in cardiac surgery.

While our sample was evenly distributed throughout Saudi Arabia's western region, other parts of the country are still unrepresented, which poses some limitations to our study. As a result, our findings cannot be generalized completely. Since we used a cross-sectional study design and a self-reported approach, another limitation is the potential for social bias.

## Conclusions

By all counts, and with the proven results of our study, this study showed that approximately 75.2% of students indicated that their knowledge and perception of cardiac surgery was not very satisfactory. 52.3% of medical students showed that cardiac surgery may not consider a career option due to their poor understanding of the importance of this specialty and its future opportunities. About 88.9% of students have never done a rotation in cardiac surgery, The findings of our study highlight the value of using innovative and targeted learning methods for medical students to enhance their knowledge and pique their interest in cardiac surgery since it was evident that there was a misperception regarding the extent of cases dealt with by cardiac surgery as opposed to other surgical subspecialists.
